# Fabrication of Porous Gold Film Using Graphene Oxide as a Sacrificial Layer

**DOI:** 10.3390/ma12142305

**Published:** 2019-07-18

**Authors:** Anas Alazzam, Nahla Alamoodi, Mohammad Abutayeh, Ion Stiharu, Vahé Nerguizian

**Affiliations:** 1 Department of Mechanical Engineering, Khalifa University, Abu Dhabi 127788, UAE; 2 Department of Electrical Engineering, École de Technologie Supérieure, Montreal, QC H3C 1K3, Canada; 3 Department of Chemical Engineering, Khalifa University, Abu Dhabi 127788, UAE; 4 Mechanical Engineering Department, Arkansas State University, Jonesboro, AR 72401, USA; 5 Department of Mechanical and Industrial Engineering, Concordia University, Montreal, QC H3G 1M8, Canada

**Keywords:** porous, gold porous, graphene oxide, metal porous, porous film, gold porous film, dielectrophoresis

## Abstract

An original and simple fabrication process to produce thin porous metal films on selected substrates is reported. The fabrication process includes the deposition of a thin layer of gold on a substrate, spin coating of a graphene oxide dispersion, etching the gold film through the graphene oxide layer, and removing the graphene oxide layer. The porosity of the thin gold film is controlled by varying the etching time, the thickness of the gold film, and the concentration of the graphene oxide dispersion. Images by scanning electron and metallurgical microscopes show a continuous gold film with random porosity formed on the substrate with a porosity size ranging between hundreds of nanometers to tens of micrometers. This general approach enables the fabrication of porous metal films using conventional microfabrication techniques. The proposed process is implemented to fabricate electrodes with patterned porosity that are used in a microfluidic system to manipulate living cells under dielectrophoresis. Porous electrodes are found to enhance the magnitude and spatial distribution of the dielectrophoretic force.

## 1. Introduction

Porous metal films have drawn a great deal of attention during the last few decades due to their unique properties, such as: high specific surface area, enhanced catalytic activity, stability, mechanical properties, and high electrical conductivity [1,2,3,4,5]. Gold porous films, in particular, are increasingly used in a wide range of applications including sensors, catalysts, transistors, solar cells, and biological operations [6,7,8,9,10,11]. Each of these applications requires a particular morphology and a specific pore size and structure. The adapted gold film fabrication technique has a direct impact on these parameters. Several studies have reported various synthesizing methods of porous gold films along with their applications [12,13]. These synthesizing methods include electrochemical de-alloying [14,15], sputtering [16,17], electron beam evaporation [18], and electrochemical deposition [19,20]. De-alloying of Ag-Au leaves yields very thin porous gold films that are very difficult to handle and extremely challenging to transfer to substrates, thus rendering the method unsuitable for standard photolithography processes [21,22]. The sputter deposition methods for synthesizing porous gold films are either based on patterned substrate or a combination of de-alloying and a patterned substrate [1,12]. The method is compatible with different microfabrication techniques but requires an advanced and costly lithography apparatus to pattern the pores of the gold film. The electron beam evaporation synthesizing method is also suitable for different microfabrication techniques but it requires complex, advanced, and costly equipment. The electrochemical deposition is one of the most utilized synthesizing methods to produce porous gold films where several approaches have been used to synthesize gold films using it. Most of these approaches require a conductive substrate, which makes the process unsuitable for microfabrication when using glass or polymer substrates. Moreover, additives in the electrochemical bath might be required to control the morphology and roughness of the porous gold film.

In this paper, a novel and low-cost approach to fabricate porous metal film on substrates is introduced. This simple method involves creating a layer of the desired metal with a specific thickness on the substrate, coating the layer with graphene oxide (GO), etching of the metal layer through the GO layer, and finally removing the GO layer from the surface of the metal. The method is suitable for microfabrication and can be used on conductive and nonconductive substrates. Specifically, this approach is used to synthesize porous gold films on Cyclic Olefin Copolymer (COC) substrates. The porosity and the pore size can be controlled by altering the concentration of the GO dispersion, etching time, and thickness of the gold film. This method can also be applied for depositing a thin gold film on glass substrates with an additional step to enhance the adhesion of the gold film to the glass. Adhesion is not an issue when gold is deposited directly onto COC because of the stable bond formed between them. The reported method is simple, cost-effective, and repeatable; furthermore, no such porous gold film fabrication method has been reported in the literature.

## 2. Materials and Instrumentations

The materials used are Graphene Oxide (Sigma Aldrich, St. Louis, MO, USA) and a gold etchant (TechniEtchTM ACI2 from MicroChemicals GmbH, Ulm, Germany). The gold deposition is achieved by a sputter (Q300 TT from Quorum Technologies, Lewes, UK), the GO is sonicated using a Branson sonicator (Thomas Scientific, Swedesboro, NJ, USA) and is dispersed using a spin coater (WS650Hzb-23NPP UD-3 from Laurell Technologies Corporation, North Wales, PA, USA). The porosity of the gold film is visualized using Scanning Electron Microscopy (JEOL JSM-7610F from Jeol Ltd., Tokyo, Japan) and a metallurgical microscope (Zeiss, Jena, Germany), and the wafers used are made of Cyclic Olefin Copolymer substrates (SIRRIS.be, Brussel, Belgium). 

### Method

**Figure 1:**
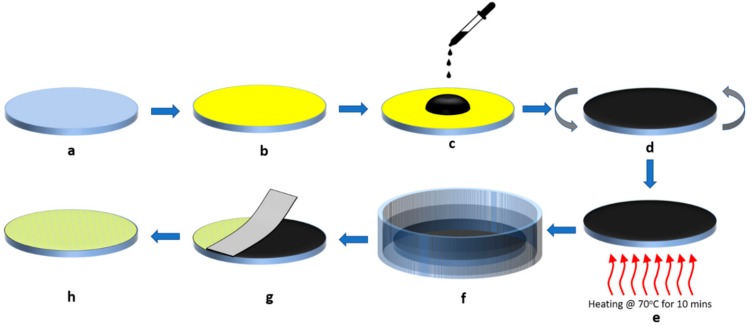
Schematic illustration of the fabrication process for porous gold thin film: (**a**) cleaning the Cyclic Olefin Copolymer (COC) wafer; (**b**) deposition of gold film on the wafer; (**c**) dispersion of the graphene oxide (GO) layer on top of the gold film; (**d**) spinning the wafer for 30 s; (**e**) heating the wafer at 70 °C for 10 minutes; (**f**) etching the gold film; (**g**) stripping-off the GO layer using an adhesive tape; (**h**) illustration of the porous gold thin film on top of the COC wafer.

A schematic representation for the synthesizing process of thin porous gold film on COC wafer is shown in Figure 1. Initially, contaminants on the COC wafer are removed by washing the COC wafer and immersing it in an isopropanol bath and sonicating it for ten minutes. The wafer is then dried with compressed dry air and baked at 70 °C for ten minutes to drive off the remaining Isopropanol (Figure 1a). Next, a thin film of gold is deposited on top of the COC wafer using a sputter (Figure 1b). 

Following the gold deposition, a layer of randomly distributed GO flakes on top of the gold film is required. GO dispersed in water is sonicated and then few drops are deposited on top of the gold film using a pipette dropper (Figure 1c). Next, the GO layer is uniformly distributed on top of the wafer by spinning the wafer at 4000 rpm for 30 s (Figure 1d). The wafer is then baked at 70 °C for 10 min to evaporate the water and improve the GO adhesion on the gold film as shown in Figure 1e. The following step is the wet etching of the gold film through the GO layer. The GO-gold coated COC wafer is immersed in the gold etchant for a specific amount of time to create pores according to the exposed surfaces of the gold film to the etchant (Figure 1f). The contact area between the etchant and the gold film is controlled by the intensity of GO flakes present on top of the gold film. Adding more GO flakes by reducing the spinning speed or by using higher GO concentrations reduces this contact area. The etching time will depend on the desired porosity as will be discussed in the next section. The etching process is ceased by removing the wafer from the etchant and washing it in deionized water several times.

The last step in the fabrication process is to liftoff the GO layer. After drying the etched wafer with compressed air, the GO flakes are removed using an adhesive tape leaving a porous thin film on the wafer as shown in Figure 1g. Finally, the gold film is further cleaned to remove any GO traces by sonicating the wafer in an isopropanol bath for ten minutes. A schematic of the finished product of a thin porous gold film on top of a COC wafer is shown in Figure 1h.

## 3. Results and Discussion

### 3.1. Characterization 

**Figure 2:**
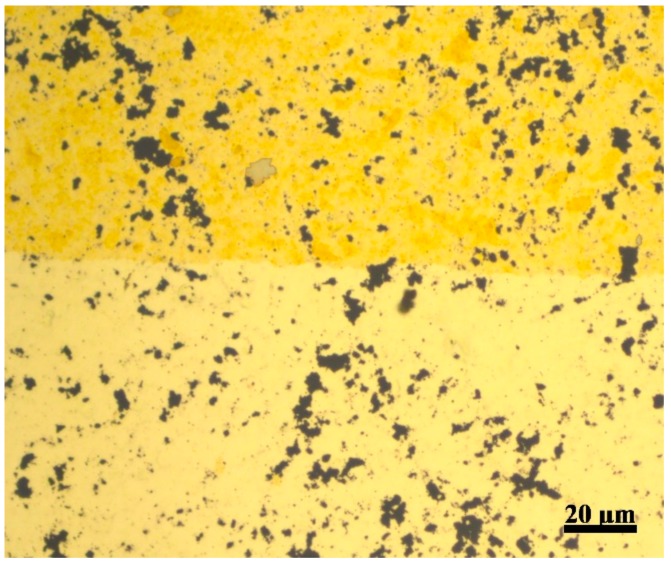
Porous gold film on a COC wafer. The GO layer is removed from the lower side using an adhesive tape.

In the illustrated fabrication method, GO dispersion is used as a random patterning layer on top of the gold film. The advantage of using GO in this application is due to several factors. First, GO is physically inert to the gold etchant; the etchant does not attack the GO flakes on top of the gold film or weakens the GO adhesiveness on the gold film. Second, the GO flakes are two-dimensional [23] which results in a large and uniform coverage area per flake compared to dispersions with spherically shaped dispersed inert materials. Third, GO can be removed in a simple procedure without the use of expensive solvents and additional complex cleaning steps. Figure 2 is an illustrative image of a porous gold film deposited on top of a COC wafer with a partially removed GO layer using adhesive tape. The image is taken using a metallurgical microscope, the lower side of the image shows the final configuration of the gold film with the GO layer removed. 

**Figure 3:**
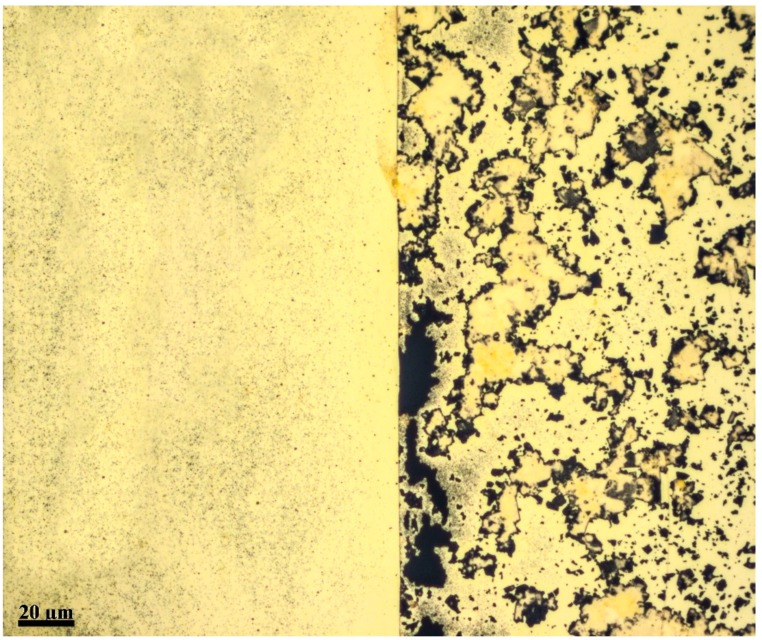
Etching of thin gold film over COC wafer. The left side of the figure shows uniform etching of gold, while the right side, coated with GO layer, shows a porous gold thin film.

**Figure 4:**
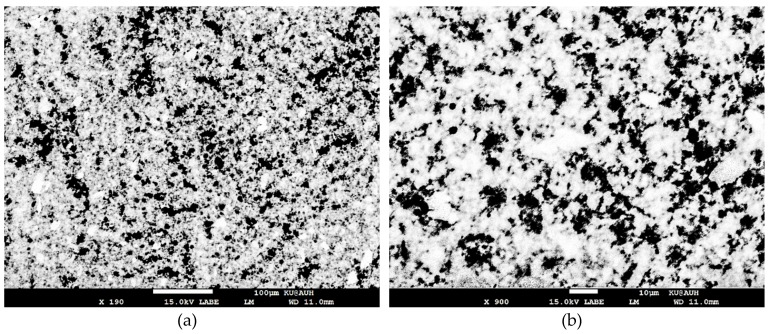
SEM images at different magnifications of 800 nm thickness porous gold film fabricated with 4 mg/mL GO and etched for 60 s. (**a**) ×190 magnification; (**b**) ×900 magnification.

The mechanism of forming porous gold film using GO is based on creating areas on the gold film that are more susceptible to etching. The GO layer acts as a passive film protecting the gold film from etching. Since the GO layer is not uniform, i.e., there are gaps between the GO flakes and there are defects within the flakes, the boundaries of the flakes, whether it is a single flake or an aggregation of flakes, creates an environment that is more likely to be attacked by the etchant. As a result, etches are initiated along the boundary of the flakes and grow deeper into the gold film at a faster rate than the areas that experiences uniform etching. Figure 3 demonstrates the difference in the etching process for a gold thin film coated with GO (right) and without coating (left). The area on the left shows uniform etching as indicated by the uniform light shade that appears through the gold film, while the area on the right shows deep etches (shown in black) that are formed along the boundaries of the GO dispersion. A scanning electron microscope (SEM) images of the gold film are shown in Figure 4. The dark areas represent the areas on the COC wafer where the gold film was etched away. This figure along with Figure 3 confirms that the gold film is a porous film with random pore sizes in range of hundreds of nanometers to few micrometers.

**Figure 5:**
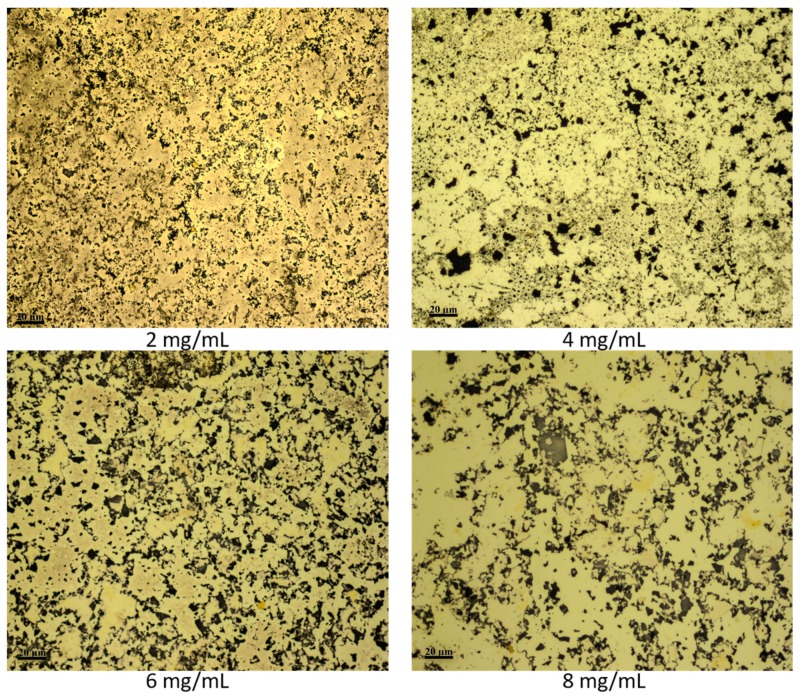
Etching of thin gold film over COC wafer with different concentrations of GO. The concentration of the GO for each sample is shown in the figure. All samples were etched for 15 s. All microscopic images were taken at the same magnification.

The gold film porosity was then characterized by finding the pore density, pore spatial distribution, and pore size distribution. The factors investigated were the concentration of the GO dispersion, etching time, and the thickness of the gold film. The GO concentration was varied to investigate its effect on the pore density and on the pore spatial distribution. Four concentrations were used, 2, 4, 6 and 8 mg/mL and the etching time was set to 15 s. The pore density was calculated as the ratio of the number of pores per surface area where the number of pores was found using the image-processing software FIJI (ImageJ 1.52h). The pore spatial distribution was approximated using the nearest neighboring distance method, which measures the nearest distance between the centroids of neighboring pores. Figure 5 demonstrates the effect of changing GO concentration on the pore density and the pore spatial distribution of the gold film. The pore density of the film is 0.1, 0.08, 0.06, and 0.04 pores/µm^2^ for the GO concentrations of 2, 4, 6, and 8 mg/mL, respectively. The decrease in the pore density as GO concentration increases is related to the number of flakes deposited on the surface of the gold film and on their orientation; whether it is a single layer deposited or multiple flakes stacked on top of each other. Since the GO layer over the gold film was formed at the same spinning speed for all GO dispersions, it is expected that at high concentration of GO dispersion, 8 mg/mL, the amount of flakes dispersed on the gold film is high and more stacked than that at lower concentrations. Therefore, due to the lower amount of GO flakes and fewer flakes stacking at a concentration of 2 mg/mL the pore density is high compared to the density at a GO concentration of 8 mg/mL.

**Figure 6:**
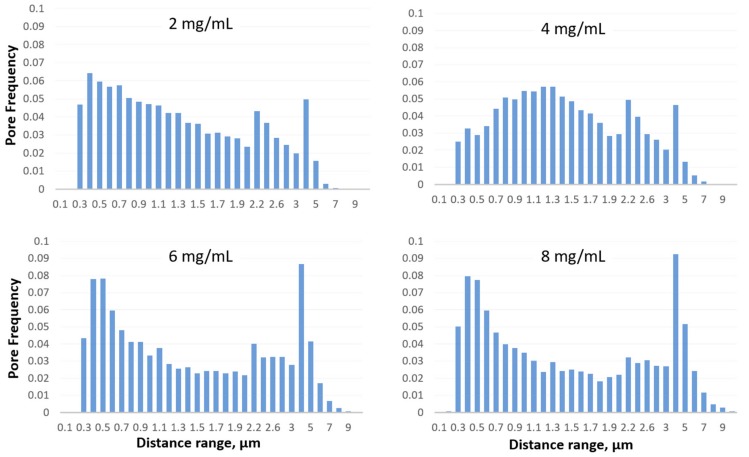
Pore spatial distribution of gold film at different GO concentrations.

The pore spatial distributions at different concentrations of GO dispersions are illustrated in Figure 6. For each GO concentration, several microscopic images reflecting different locations on the thin gold film were analyzed to find the pores nearest neighboring distance from which the distributions were obtained. The distributions for all the cases of the GO concentrations indicate pore distances that range from 0.1 to 10 µm. At low concentration of GO dispersion, 2 mg/mL, the pore frequency decreases nearly uniformly with high pore frequency at distances ranging from 0.3–1.3 µm and low pore frequency at distances greater than 4 µm. The spatial distributions at higher concentrations of GO dispersion, 6 and 8 mg/mL, show nearly an identical and peculiar trend. It is expected that at higher concentrations of GO dispersions the frequency of the pores will be highest at large distance ranges. However, the distributions show a maximum pore frequency of 18% at distance ranges greater than 5 µm and a frequency of approximately 43% at distance ranges smaller than 1 µm. This is possibly due to the stacking of the GO flakes at high concentrations. Stacking can result in complete and partial overlapping of the GO flakes; partial overlapping will create microscopic areas between the edges of the aggregated flakes that are exposed to the etchant, while complete overlapping results in larger areas of the gold film that are covered. Therefore, the areas created between the boundaries of the partially overlapping flakes result in pores that are close to each other, while the completely overlapping GO flakes result in pores that are away from each other with distance ranges greater than 5 µm. Another possible explanation is that at high concentrations of GO dispersions, the adhesion between the GO flakes as they stack over each other on the gold film may be stronger than the adhesion of a single layer of GO flakes on the film. This causes some of the GO stacked flakes to peel off as the adhesion on the gold film is disturbed by the etchant. Hence, increasing the concentration of GO dispersions will result in a pore spatial distribution close to that at low concentration, however, with less uniformity. 

**Figure 7:**
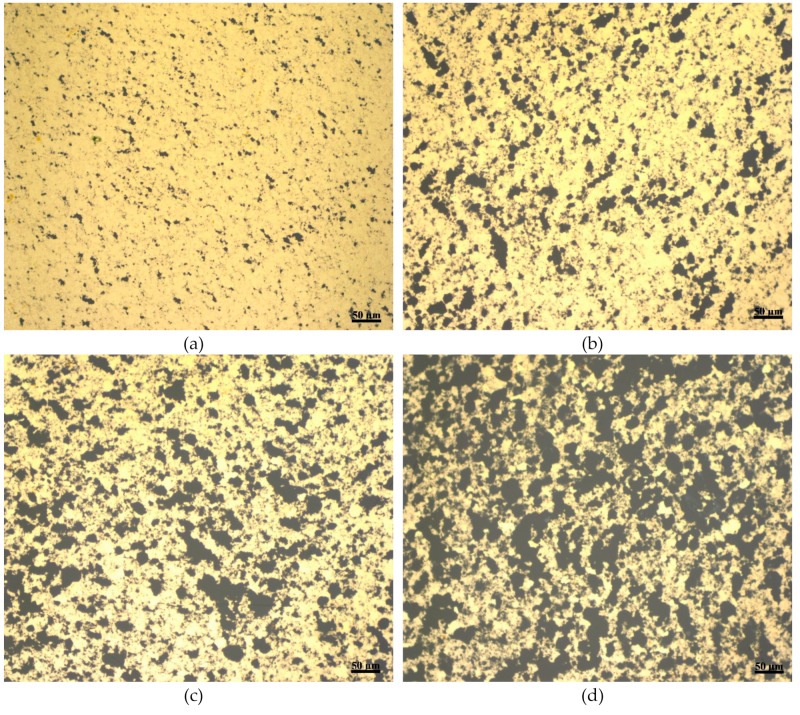
Microscopic images for porous gold film fabricated with different etching times: (**a**) 15 s (**b**) 30 s (**c**) 45 s, (**d**) 60 s, where the thickness of the gold film for all wafers is ~800 nm and the concentration of the GO dispersion is 4 mg/mL.

As the etching time has a direct impact on the sizes and shapes of the pores, the effect of the etching time on the porosity of the gold film is then investigated. Figure 7 demonstrates the effect of increasing the etching time on the pores size of the film. All images were taken with a metallurgical microscope and at the same magnification. The microscopic images are for four different wafers fabricated following the same procedure illustrated in Figure 1 but with different etching times. The etching times for the gold films in Figure 7a–d are 15, 30, 45, and 60 s, respectively. The thickness of the gold film for all wafers is ~800 nm and the concentration of the GO dispersion is 4 mg/mL. While Figure 7 indicates that the pore size increases with etching time, the size increase is a function of several variables; the first variable is the exposed gold film areas between the flakes, the second one is the under-etching of the gold film that occurs beneath the boundaries of the GO flakes, and the third one is the merging of growing pores into single larger pores. Other factors as temperature or etchant concentration have not been looked up to in this study. As discussed above, the exposed areas between the flakes are dictated by and can be controlled using different concentrations of GO dispersions. The under-etching of the gold film and the merging of the growing pores depend on the etching time as well as the thickness of the gold film. Increasing the etching time will cause the size of the individual pores to increase and the merging of growing pores to single pores approaching the size of the exposed areas between the flakes as evident from Figure 7, and will allow for etching to occur beneath the GO flakes on the gold surface. Longer etching periods causes the gold to lose its unity as a single film creating isolated islands of gold film on the top of the wafer as shown in Figure 7d. The porosity of the gold film is found to be 8.6%, 23.7%, 35.9%, and 46.3% for etching times 15, 30, 45, and 60 s, respectively. 

**Figure 8:**
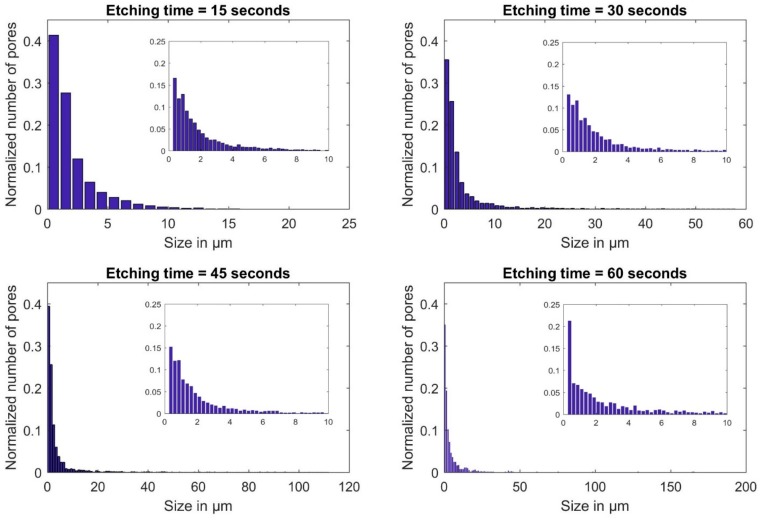
Distribution of pores in the gold film at different etching rates, where the thickness of the gold film for all wafers is ~800 nm and the concentration of the GO dispersion is 4 mg/mL.

The effect of etching time on the porosity of the gold film is further analyzed by studying the sizes of pores at different places of the wafer. Ten microscopic images taken at different places of the porous gold film are used to calculate the sizes and numbers of pores using FIJI software (ImageJ 1.52h). Figure 8 shows the statistical analysis of pore size in the porous gold film at different etching times where the insets show the size distribution for pores sizes up to 10 µm. It is evident from Figure 8 that increasing the etching time has little effect on pores less than 1 µm in size. Furthermore, at etching time of 60 s the number of pores with size less than 0.5 µm is almost twice as much as the number of pores at etching time of 30 s for the same size, this could be due to the repositioning of the GO flakes during the etching process. As the etching time increases, the pores initiated at the boundaries of the GO flakes grow in size travelling partially underneath the flakes. The void created under the flakes then causes them to reposition themselves creating new sites for pore formation. 

**Figure 9:**
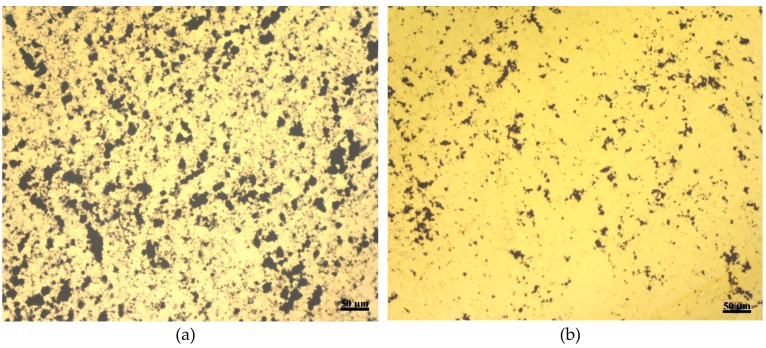
Two microscopic images for porous gold film with film thickness of: (**a**) ~800 nm (**b**) ~1400 nm where the concentration of the GO dispersion is 4 mg/mL and the etching time for the gold film is 30 s.

Since the gold film thickness is another important factor for controlling the pores size, its effect is analyzed next. Gold films were deposited on COC wafers using a sputter with their thickness controlled by altering the sputtering time. Figure 9 shows two microscopic images of porous gold films on two different COC wafers at the same magnification. The porous gold films on both wafers were fabricated following the same procedure illustrated in Figure 1 but with two different film thicknesses: ~800 nm shown in Figure 9a and ~1400 nm shown in Figure 9b. The concentration of the GO dispersion and the etching time for both wafers are 4 mg/mL and 30 s, respectively. The speed of pores development in the gold film corresponds to the thickness of the same. It is clear from the two images in Figure 9 that increasing the thickness of the gold film results in less porosity. On one hand the thickness of the gold film was found to have a minor effect on the number of pores that are less than 2 µm in size, about 60% of the pores are in that range for both thicknesses, 800 nm and 1400 nm, and on the other hand, increasing the thickness of the gold film has significantly decreased the pores with sizes greater than 2 µm. The porosity and the average pores size in Figure 9a,b are 26%, 8%, 3.1 µm, and 2.6 µm respectively. 

### 3.2. Dielectrophoresis as an Application

The applications of porous gold film range from biological and chemical sensing to large-scale engineering use. In this work, we investigated the use of gold porous electrodes for manipulating of living cells under dielectrophoresis (DEP). A thin film of porous gold electrodes fabricated according to the procedure discussed above was used to create an inhomogeneous electric field to attract red blood cells (RBCs). DEP is a label-free electrokinetic phenomenon used for manipulating cells and particles under non-uniform electric field [24,25,26]. It is defined as the motion of polarizable particle suspended in conductive medium under the effect of non-uniform electric field. Dielectrophoresis force depends on several factors as stated in the equation below:

$$F_{DEP}=2\pi \varepsilon_{m}r_{p}{}^{3}Re\left[f_{CM}\right]\nabla {\left|E_{rms}\right|}^{2}$$

where $\varepsilon_{m}$ is the permittivity of the medium, $r_{p}$ is the radius of the particle, $Re\left[f_{CM}\right]$ is the real part of Clausius-Mossotti factor, ∇ is the gradient operator, and $E_{rms}$ represents root mean square value of the Electric Field. The real part of Clausius-Mossotti factor ranges from −0.5 to 1 and it depends on the applied frequency of the AC signal and the type of cells. 

**Figure 10:**
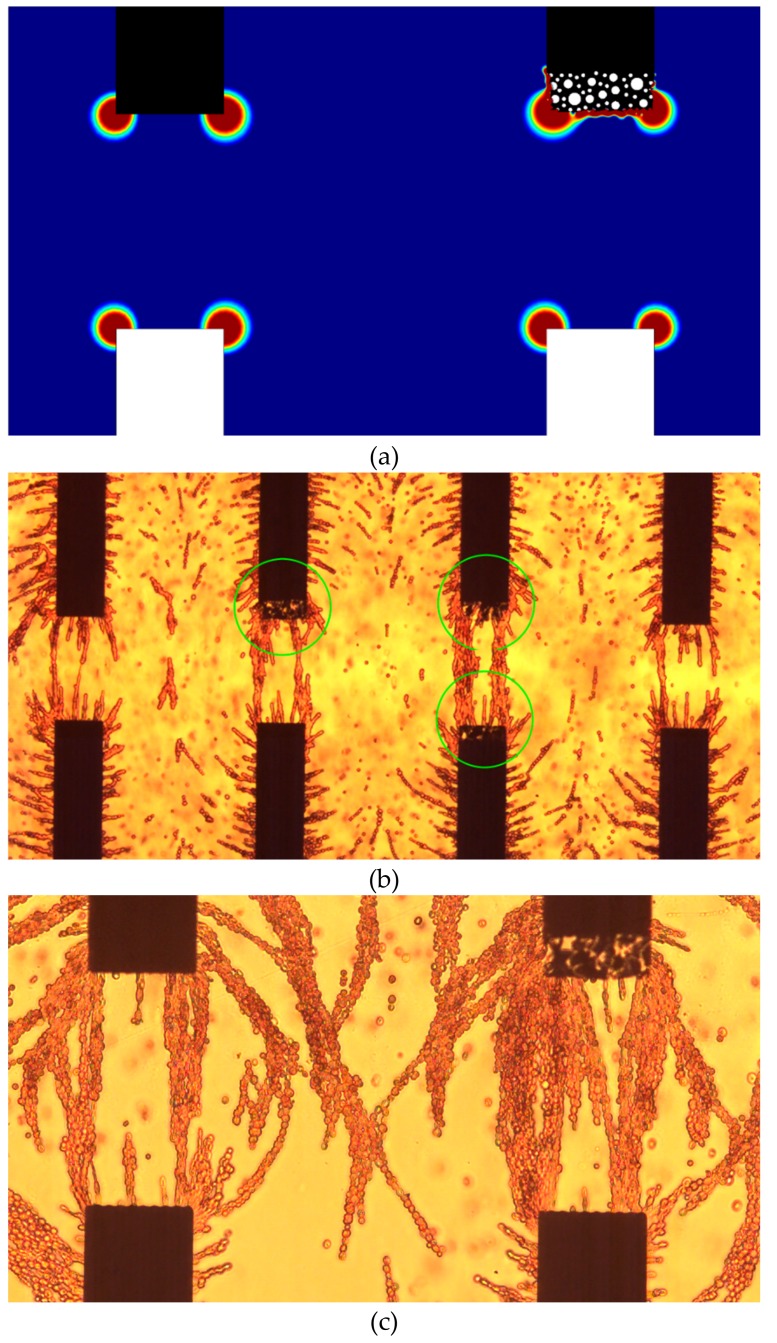
(**a**) The contour plot for the term $\nabla {\left|E_{rms}\right|}^{2}$ between solid-solid and porous-solid electrodes. The white electrodes are connected to ground. (**b**) The accumulation of red blood cells due to positive dielectrophoresis. Circles are added to indicate the porous electrodes. (**c**) The accumulation of RBCs between the electrodes at higher magnifications. The width of the electrodes is 100 µm, the applied voltage is 5 volts, and the frequency is 1 MHz for the three subfigures.

DEP force could be negative where the particles are repelled by the high gradient of the electric field or positive where the particles are attracted toward the high gradient of the electric field. Both phenomena are termed negative DEP (nDEP) and positive DEP (pDEP), respectively [27,28,29]. DEP has been extensively used in microfluidic systems for manipulating of particles and cells. The magnitude of the DEP force depends on the electrical properties, size, and shape of the particle, conductivity of the medium, and the frequency, non-uniformity, and amplitude of the AC signal [30]. As the force is linearly proportional to the term $\nabla {\left|E_{rms}\right|}^{2}$, one approach to enhance manipulation of cells using DEP is by increasing the spatial non-uniformity of the electric field [29,31,32]. Increasing the porosity at the outer layer of electrodes creates more edges. The charge density in these edges is always larger due to “edge effect”. Thus, the electric field near the edges of the electrodes is always larger than the electric field between them. Figure 10a shows the contour plot for $\nabla {\left|E_{rms}\right|}^{2}$ between solid-solid and solid-porous electrodes. It is evident from this figure that porosity at the edges of electrodes improves both, the DEP force magnitude and the spatial distribution. 

The effect of electrodes porosity on DEP was experimentally investigated. Electrodes were initially patterned using standard microfabrication processes that include sputtering, spin coating, etching, and photolithography. The tips of the electrodes were then selectively made porous using photoresist as masking material. The thickness of the gold film was 800 nm and the concentration of the GO dispersion was 4 mg/mL. The electrodes were etched for 30 s to create porosity at the tips. Figure 10b shows the aggregation of RBCs using porous and solid electrodes. The RBCs were suspended in sucrose-dextrose isotonic medium. The experimental setup and medium preparation were previously reported [24,29]. It is clear from Figure 10b that porous electrodes generate higher DEP force and attract more cells. The accumulation of cells due to pDEP was found to be higher between porous-porous electrodes and on the porous electrodes when porous-solid electrodes were used. Figure 10c shows the accumulation of RBCs between the electrodes at a higher magnification.

## 4. Conclusions

A novel and low-cost method to fabricate porous thin gold film on polymer substrates is proposed. The porous film is created by etching the gold film through a layer of GO. This method has been demonstrated by fabricating porous films of gold on top of COC substrates with different porosities. The fabrication process includes conventional microfabrication techniques such as sputtering, spin coating, wet etching, and GO liftoff. The utilization of the GO results in the nucleation of the pores along the boundaries of the flakes. The porosity of the gold film could be controlled by selecting an optimum thickness of the gold film, the concentration of the GO dispersion, and the etching time of the gold film. Smaller pore sizes could be achieved if the thickness of the gold film is increased, or the etching time of the gold film is reduced. A more uniform porous film could be achieved by using low concentrations of GO dispersion. The porous gold film could be used for a broad range of applications and the reported fabrication method is applicable for conventional microfabrication techniques. Electrodes with patterned porosity using GO were fabricated and used to attract red blood cells under dielectrophoresis. Porous electrodes were found to enhance the magnitude and spatial distribution of the DEP force. The process layout is given for gold electrodes; however, it is applicable to create any metallic porous films.
